# Navigating self‐doubt in modern medicine

**DOI:** 10.1002/jhm.13469

**Published:** 2024-07-24

**Authors:** Lawrence Kwon

**Affiliations:** ^1^ Department of Nephrology Westchester Medical Center Advanced Physician Services, Mid‐Hudson Regional Hospital Poughkeepsie New York USA

My patient, who I'll call Jay, confided in me, unease gripping him tightly. He recounted a dream from over a decade ago that had left an indelible mark. In that dream, he saw his early demise, and now, in his early 50s, he felt that ominous prediction slowly unfolding.

He appeared disheveled with loose sweatpants chosen for comfort, as any contact with his left hip and leg caused searing pain. I noted his thick glasses and impaired hearing, which necessitated my sitting to his left and almost shouting at him. He complained of a persistent headache in his right temple, with occasional throbbing behind his right eye. His left foot felt numb, and he described tingling around his lip and chin. He also mentioned a sensation of detachment from his own body.

He suffered a cerebellar stroke 2 years ago. Since then, he had an increasing serum creatinine and worsening proteinuria. We attributed those abnormalities to his diabetes. He had abandoned metformin due to its adverse effects but continued taking his long‐acting insulin daily.

I couldn't accept that all his symptoms could solely be attributed to diabetes; his A1c was a modest 7.4%. I next conducted a hurried internet search for his symptoms. And there it was: Fabry disease. His life had been troubled by fatigue, unrelenting nausea, and vomiting that kept him bedridden for hours. He consumed nearly 6 quarts of water daily and mentioned a recent visit for cataracts. When I asked about his perspiration, he smiled, noting that he hadn't sweated at all. My heart raced.

I needed a thorough family history, but his recollection was poor. He was uncertain about his father and could provide little information about his late mother, who had suffered from multiple strokes and was diagnosed with multiple sclerosis. Had her symptoms been neurologic manifestations of Fabry disease? The strain of articulating my questions loudly and repeatedly made me reflect on how little I knew about my own family's medical history.

After almost 2 h of conversation, my initial excitement began to wane, replaced by a sense of fatigue and self‐doubt. I realized that I had been dedicating too much time to this investigation when other patients needed my attention. Modern medicine did not seem to leave much time to embrace curiosity.

As I exited the conference room, the nurses noticed the extended duration of my conversation. “Wow, you were in there for quite a while,” they remarked. I shared my thoughts, explaining that this case went beyond uncontrolled diabetes. I reflected on the saying that we all learn in medical school, “When you hear hoofbeats, think horses, not zebras.” As I chatted with the nurses, I noticed the patient wandering around, his demeanor reflective, a sobering moment that grounded me back to earth.

I confessed to the patient that I had never encountered Fabry disease before and that it would take weeks to know the result of a diagnostic test for alpha‐galactosidase A enzyme activity. He accepted this, acknowledging that he needed a new perspective on his health. I told him that if he did have the disease, I would help him seek a second opinion and expert advice. He reassured me that was satisfactory and thanked me for spending so much time with him.

The results finally arrived: normal alpha‐galactosidase A enzyme activity. This result made Fabry disease extremely unlikely. My heart sank. I was devastated. With the negative report, my dedication to his case waned, and I found myself caring less about his condition. The weight of disappointment settled in. Should I order the test again to ensure it wasn't a false negative? I grappled with the notion that having a diagnosis wouldn't alter the management; why should I dwell on it? Even if it were Fabry disease, it was likely too late for enzyme therapy to make a difference in his condition.

I had poured an immense amount of time and effort into this case. I wondered if my relentless pursuit was more about proving myself than serving the patient's best interests. When receiving the result, why was my first thought about me and not the patient? This experience stirred memories of my academic challenges, poor grades, barely passing USMLEs and board examinations, and difficulties during clinical years due to my meticulous note‐writing. Why did I spend so much time with this patient? Why couldn't I just write a standard note saying that this was nothing more than complications of diabetes and briefly mention Fabry disease? I felt a sense of naiveté. Had my desire for a particular diagnosis led me to ask leading questions? Who asks about a patient's ability to sweat?

Challenges and disappointments in my medical career have made me constantly feel the need to prove my skills with jealousy toward others' successes and the tendency to project my inner struggles onto external circumstances. Perhaps being overly analytical, coupled with the frustration of not achieving small victories like making an impressive and novel diagnosis in this case, has exacted a greater mental and emotional toll than I had anticipated. Maintaining self‐compassion becomes challenging when so much of my identity is wrapped up in being considered an exemplary physician.

I am beginning to understand that the relentless pursuit of perfection not only drives one mad but also subtly prioritizes my need for validation over the patient's needs. Self‐doubt is inherent in being a doctor, and acknowledging this doubt fosters empathy toward myself and my patients. Medicine is rarely straightforward, and the effort to deeply understand a condition is just as valuable as the outcome. This perseverance enables us to find joy in our work, engaging deeply with both the science and the soul of medicine. It allows us to connect with another human being in a way that transcends mere knowledge acquisition and redefines our triumphs and setbacks.

I called the patient a few days after the enzyme test results had come in, bearing the news that my hypothesis was wrong. By that time, he had undergone coronary artery bypass surgery and initiated hemodialysis. His response to me was unexpectedly gracious; he reassured me that I had done my best and was content with that. He was most grateful that I spent much time listening to him and trying to understand his condition. I was moved to tears and overcome with gratitude as I thanked him.

## CONFLICT OF INTEREST STATEMENT

The author declares no conflict of interest.

